# Instagram Posts Promoting Colorectal Cancer Awareness: Content Analysis of Themes and Engagement During Colorectal Cancer Awareness Month

**DOI:** 10.2196/63344

**Published:** 2025-02-19

**Authors:** Aditi Srivastava, Jim P Stimpson

**Affiliations:** 1 Frisco High School, Frisco, TX, United States; 2 Department of Health Economics, Systems, and Policy, Peter O'Donnell Jr. School of Public Health, The University of Texas Southwestern Medical Center, 5323 Harry Hines Blvd, Dallas, TX, 75390, United States, 1 214-645-2567

**Keywords:** social media, colorectal neoplasms, early detection of cancer, public health, health inequities, harnessing, Instagram, colorectal cancer, colorectal cancer awareness, content analysis, cancer-related deaths, detection, screening, mortality, post, early detection

## Abstract

**Background:**

Colorectal cancer (CRC) is a leading cause of cancer-related deaths worldwide, with early detection and screening being critical for reducing mortality. Social media platforms like Instagram offer a unique opportunity to raise awareness about CRC, particularly during designated awareness months. However, there is limited research on the effectiveness of CRC-related content on Instagram.

**Objective:**

This study aims to examine how Instagram is used to raise awareness about CRC during Colorectal Cancer Awareness Month by analyzing the thematic content and engagement metrics of related posts. The research seeks to identify the prevalent themes, assess audience interaction with these messages, and highlight areas for improvement in leveraging Instagram as a tool for cancer awareness campaigns.

**Methods:**

A total of 150 Instagram posts were collected based on their use of specific hashtags related to CRC awareness (#colorectalcancer, #colorectalcancerawareness, #colorectalcancerawarenessmonth) during March 2024. The text and images in the posts were categorized into themes such as screening and early detection, symptoms, general awareness, risk factors, individual’s experiences, representation of racial and ethnic minoritized communities, and representation of women. Engagement metrics, including the number of likes and comments, were also analyzed. Two researchers independently coded the posts, achieving high interrater reliability (Cohen κ=0.93).

**Results:**

Organizational accounts were more active, contributing 82% (n=123) of the 150 posts, compared to 18% (n=27) from individual users. The most frequently mentioned theme was screening and early detection, which made up 37.3% (n=56) of all posts. General awareness came in second at 19.3% (n=29), and risk factors came in third at 12% (n=18). Posts about individual experiences and general awareness received the highest engagement, indicating the effectiveness of personal narratives and broad informational content. Themes related to symptoms and representation of racial and ethnic minoritized communities and women were underrepresented.

**Conclusions:**

This study highlights the potential of Instagram as a platform for promoting CRC awareness, particularly through posts about screening and early detection and personal experiences. However, there is a need for more inclusive and diverse content to ensure a broader reach and impact.

## Introduction

Colorectal cancer (CRC) remains one of the most prevalent and deadly forms of cancer worldwide [1,2]. Despite the clear benefits of early detection and screening for reducing mortality rates, many individuals remain unaware of the importance of regular screenings and the risk factors associated with CRC [3-5]. March is designated as Colorectal Cancer Awareness Month, a period dedicated to increasing public knowledge about CRC, promoting early detection and screening, and ultimately reducing the incidence and mortality of this disease [6]. During this month, various health organizations, advocacy groups, and individuals spread awareness through different communication channels, including social media.

Instagram, a visual-centric platform with over a billion users, offers a unique opportunity to disseminate health information widely and engage with a diverse audience [7-9]. The platform’s use of hashtags allows for the categorization and amplification of posts, making it easier for users to find and share relevant content. In the context of health awareness campaigns, hashtags such as #colorectalcancer, #colorectalcancerawareness, and #colorectalcancerawarenessmonth can help consolidate posts under a common theme, enhancing the visibility of key messages [10].

Instagram has been shown to be instrumental in disseminating cancer prevention material. A study of Brazilian Instagram accounts during a nationwide cervical cancer campaign revealed an emphasis on secondary prevention measures, such as Pap screenings, and primary prevention strategies, like the human papillomavirus (HPV) vaccine, received less attention [11]. A descriptive study of Instagram posts related to breast cancer found that the most common posts were individual stories or posts discussing treatment [12]. A separate investigation examined the portrayal of skin cancer narratives on Instagram and underscored the platform’s potential for sharing personal experiences and fostering prevention efforts and awareness [13]. A study that searched specific hashtags on Instagram for posts about acute myeloid leukemia found that the most frequently posted type of content by patients were health updates, and a quarter of these posts described symptom experiences [14]. Finally, a content analysis was conducted on information related to CRC on Instagram using the hashtag #colorectalcancer and found the most common posts supported someone with CRC, provided an individual story, or discussed treatment [15]. Collectively, these studies highlight Instagram’s potential as a tool for raising cancer awareness and promoting preventive behaviors, while emphasizing the need for further research to identify the most impactful types of cancer prevention messaging and to optimize social media platforms for effective dissemination of cancer prevention information [16-20].

Despite the potential of Instagram to raise awareness about CRC, there is limited research on how effectively the platform is used for this purpose, particularly during dedicated cancer awareness months. This study aims to fill this gap by examining the engagement of Instagram posts related to CRC during Colorectal Cancer Awareness Month. By analyzing the engagement metrics of these posts, this research seeks to understand the current state of CRC awareness on Instagram and identify areas to improve cancer communication strategies. By analyzing Instagram posts based on specific hashtags and thematic content, the findings offer insights into the types of messages that resonate most with the audience and highlight the strengths and weaknesses of current CRC awareness efforts on social media. Ultimately, this research contributes to the broader goal of enhancing public health campaigns and increasing the effectiveness of social media as a tool for health promotion.

## Methods

### Data Collection

The data for this study were collected from Instagram, focusing specifically on public posts related to CRC published from March 1 to 31, 2024, which is Colorectal Cancer Awareness Month. These dates were chosen to capture the peak of awareness activities and messages. Using Instagram’s search functionality, the top 150 posts containing hashtags related to CRC were identified and collected. The hashtags used for this search included #colorectalcancer, #colorectalcancerawareness, #colorectalcancerawarenessmonth, and combinations of these hashtags. To ensure comprehensive coverage, a supplementary search was conducted using additional terms such as “CRC,” “Colon cancer,” and “Colorectal Cancer.” This supplementary search confirmed that the top posts identified through these terms consistently included 1 or more of the primary hashtags, indicating that the initial hashtag-based approach effectively captured relevant posts. This process minimized the risk of excluding significant content while aligning with the study’s focus on thematic content and engagement during Colorectal Cancer Awareness Month. The number of likes and comments on the posts served as a gauge of their popularity, ensuring a representative sample of both highly engaged content and content with little engagement.

Given that institutional accounts often repost the same content multiple times during awareness campaigns, we implemented a systematic approach to handle duplicate posts. If a post was identical in both text and hashtags and originated from the same account, only the first instance was included in the dataset to prevent overrepresentation. However, if a repost contained minor variations in wording or hashtag use, it was retained to capture potential differences in engagement strategies. Engagement metrics (likes and comments) were analyzed separately for each retained post, rather than aggregating them across duplicates, as each post had the potential to generate unique interactions. This approach ensured that our dataset reflected a diverse range of content while minimizing redundancy and maintaining the integrity of engagement analysis.

### Coding Process

This study employed a deductive content analysis approach to analyze both the text and images of Instagram posts related to CRC awareness. The thematic categories used for coding were predefined based on existing literature and theoretical frameworks in health communication [21-23]. The deductive approach was intentionally chosen to assess the prevalence and resonance of these theoretically supported themes, as reflected by engagement metrics (eg, likes, comments). This structured framework allowed for a systematic evaluation of whether established health communication messages were effectively disseminated through Instagram posts during Colorectal Cancer Awareness Month.

The coding framework was developed based on a review of existing literature on health communication and social media and common themes in CRC awareness [11-15]. Screening and early detection posts emphasized the importance of regular screenings and early detection methods. Symptom posts provided information on the symptoms of CRC.

General awareness posts were aimed at raising general awareness about CRC through encouraging others to post about it or stating general statistics. Risk factor posts discussed factors that increase the risk of developing CRC and, conversely, preventative lifestyle measures one can take to reduce their risk. Individuals’ experience posts shared personal stories and experiences of individuals affected by CRC. Because racial and ethnic minoritized groups and women face significant barriers to screening and care due to socioeconomic issues, limited health care access, and cultural or systemic biases, we examined posts on racial and ethnic minoritized communities and women to uncover messaging gaps and improve future awareness efforts’ reach and impact [1,3]. Therefore, the representation of racial and ethnic minoritized community posts highlighted the vulnerability of racial and ethnic minoritized communities to CRC, and the representation of women posts focused on how CRC affects women and the risks it poses. Irrelevant posts were not related to CRC or awareness activities. Finally, other posts contained relevant information about CRC but did not fit into the predefined categories.

Captions and images contained in the Instagram posts were coded to align with the predefined thematic categories. Each post was examined holistically, with its image and caption assessed collectively to determine the dominant theme. Images were categorized based on their primary visual content and captions were analyzed alongside images to provide context and ensure consistency in thematic classification. If the image and caption conveyed different themes, coders determined which element carried the primary message, prioritizing captions for text-heavy posts and images for visually dominant content. In cases where thematic classification was unclear, discrepancies were resolved through discussion between researchers to ensure consistency and accuracy in coding.

To ensure reliability and minimize bias, a systematic coding process was implemented. One researcher (AS) initially coded all posts in the dataset, applying the predefined thematic categories based on existing literature and the objectives of the study. To validate the coding and minimize potential bias, a second researcher (JPS) independently coded a random sample of 10% of the posts. Discrepancies in coding between the 2 researchers were resolved through discussion and consensus, refining the thematic framework as needed. The level of agreement between the 2 researchers for the text and image content of the posts was measured using the κ reliability statistic, which resulted in a high agreement rate of 93%. This high level of interrater reliability confirms the consistency and accuracy of the thematic classification.

### Analysis of Content and Engagement

Hashtags and post content were analyzed separately as they serve distinct functions in social media communication. Hashtag analysis was conducted to understand how users label and promote their posts within the broader discourse of CRC awareness, which helped identify trending topics, user engagement strategies, and the reach of campaign-related messaging. In contrast, analyzing the textual content (captions) and images within posts to determine the thematic focus of the message itself allowed us to assess whether the themes promoted through hashtags aligned with the actual messages in the post captions and images.

Our analysis began by describing the distribution of hashtags used for our search during Colorectal Cancer Awareness Month and the engagement of Instagram posts in terms of posts, likes, comments, and hashtag counts. Then, we described the content of the Instagram posts by hashtag, thematic category, and user profile (individual versus organizational account). This study adheres to the STROBE (Strengthening the Reporting of Observational Studies in Epidemiology) guidelines for reporting observational studies.

### Ethical Considerations

The University of Texas Southwestern Medical Center Institutional Review Board determined that the study does not meet the definition of human subjects research and therefore does not require institutional review board approval or oversight (Y1-23-0390). As the data used are publicly available on Instagram, no anonymization or deidentification was required.

## Results

This study analyzed a total of 150 Instagram posts related to CRC during Colorectal Cancer Awareness Month. These posts were categorized based on specific hashtags and the captions and image content of each post were classified into various thematic categories to understand the messaging and engagement trends.

Table 1 presents the distribution and engagement metrics of the posts across different hashtags. Posts tagged with #colorectalcancer received the highest engagement among the posts that were singularly tagged, amassing 9751 likes and 560 comments from 16 posts. This indicates a substantial interaction level, suggesting that this hashtag effectively attracts attention and engagement from users. The hashtag #colorectalcancerawarenessmonth was the most frequently used, appearing in 45 posts. These posts collectively garnered 6065 likes and 188 comments, reflecting their widespread use and moderate engagement. #colorectalcancerawareness was utilized in 31 posts, which received 3712 likes and 66 comments. This shows a lower engagement compared to the other hashtags, despite its considerable use. Posts with multiple tags were the most common, with 58 instances. These posts achieved the highest overall engagement, accumulating 17,729 likes and 120 comments. The use of multiple hashtags likely expanded the reach and visibility of the posts.

**Table 1.** Hashtag distribution and engagement of 150 Instagram posts during Colorectal Cancer Awareness Month, March 1-31, 2024.

Hashtag	Posts, n	Likes, n	Comments, n	Hashtag count, n
#colorectalcancer	16	9751	560	107
#colorectalcancerawarenessmonth	45	6065	188	132
#colorectalcancerawareness	31	3712	66	100
Multiple tags	58	17,729	120	414

Table 2 classifies the content of the Instagram posts by hashtag and thematic category. Screening and early detection was the most prevalent theme, featuring in 56 posts. This underscores a strong focus on promoting regular screenings and the importance of early detection in preventing the severity and mortality associated with CRC. General awareness was the theme in 29 posts, which highlighted efforts to spread general knowledge about CRC, including statistics and awareness activities. Risk factors were discussed in 18 posts, educating users about the various factors that increase the risk of developing CRC. Posts about the symptoms of CRC appeared in 12 instances, providing crucial information on recognizing the signs of the disease. Individual’s experiences were shared in 16 posts, offering personal narratives and testimonies from individuals affected by CRC, which can be powerful in humanizing the disease and encouraging others to take preventive actions. The representation of racial and ethnic minoritized communities and women was notably limited, with only 3 and 2 posts, respectively. This indicates a gap in addressing the specific risks and experiences of these groups, suggesting a need for more inclusive messaging. The other category included 14 posts that were relevant but did not fit into the predefined categories, reflecting a diversity of information and perspectives related to CRC. Only 1 post was classified as irrelevant, demonstrating that most posts were pertinent to the topic.

**Table 2.** Content classification of 150 Instagram posts by hashtag during Colorectal Cancer Awareness Month, March 1-31, 2024.

Hashtag	Screening or early detection (n=56), n	Symptoms (n=12), n	General awareness (n=29), n	Risk factors (n=18), n	Individual’s experience (n=16), n	Representation of racial and ethnic minority communities (n=3), n	Representation of women (n=2), n	Other (n=14), n	Irrelevant (n=1), n
#colorectalcancer (n=16)	5	1	1	1	4	0	1	2	1
#colorectalcancerawarenessmonth (n=45)	21	3	4	5	5	1	1	5	0
#colorectalcancerawareness (n=31)	13	3	6	5	2	1	0	1	0
Multiple tags (n=59)	17	5	18	7	5	1	0	6	0

Table 3 categorizes the 150 Instagram posts by user profile type—individual users (denoted as “Person”) and organizational accounts (denoted as “Organization”). The distribution of these categories was analyzed across the 2 user profile types. Organizational accounts were more active overall, contributing 82% (n=123) of the total posts, compared to 18% (n=27) from individual users. Screening and early detection emerged as the predominant category overall, with 37.3% (56/150) of all posts falling into this category. This category was significantly more common among organizational accounts, constituting 41.4% (51/123) of their posts, compared to 19% (5/27) of posts from individual users. Posts related to symptoms accounted for 8% (12/150), with a nearly equal distribution between individual and organizational accounts. The general awareness category comprised 19% (n=29) of the total posts, with individual users and organizational accounts contributing 19% (5/27) and 19.5% (24/123) of their posts, respectively. Risk factors were addressed in 12% (18/150) of the posts. Both individual users and organizational accounts showed interest in this category, with 19% (5/27) of individual users’ posts and 10.5% (13/123) of organizational accounts’ posts mentioning risks. Posts discussing individuals’ experiences accounted for 10.6% (16/150) of the total, with greater representation from individual users at 19% (5/27) compared to organizational accounts. Posts representing racial and ethnic minority communities were notably sparse, comprising only 2% (3/150) of the total posts. Individual users contributed 7% (2/27) and organizational accounts just 0.8% (1/123). Similarly, posts focusing on the representation of women were minimal, representing 1.3% (2/150) of the total posts, with no contributions from individual users and 1.6% (2/123) from organizational accounts. The other category included 9.3% (14/150) of the total posts, with individual users contributing 7% (2/27) and organizational accounts contributing 9.7% (12/123). Lastly, irrelevant posts constituted 0.6% (1/150) of the total, all from individual users at 4% (1/27), with no contributions from organizational accounts.

**Table 3.** Content classification of 150 Instagram posts by user profile during Colorectal Cancer Awareness Month, March 1-31, 2024.

	Person (n=27), n (%)	Organization (n=123), n (%)	Total (N=150), n (%)
Screening and early detection	5 (19)	51 (41.4)	56 (37.3)
Symptoms	2 (7)	10 (8.1)	12 (8)
General awareness	5 (19)	24 (19.5)	29 (19.3)
Risk factors	5 (19)	13 (10.5)	18 (12)
Individual’s experience	5 (19)	10 (8.1)	15 (10)
Representation of racial and ethnic minoritized communities	2 (7)	1 (0.8)	3 (2)
Representation of women	0 (0)	2 (1.6)	2 (1.3)
Other	2 (7)	12 (9.7)	14 (9.3)
Irrelevant	1 (4)	0 (0)	1 (0.6)

## Discussion

### Principal Findings

The goal of our research study was to explore the thematic content and engagement patterns of Instagram posts shared during Colorectal Cancer Awareness Month, with a focus on understanding how key messages about CRC prevention and awareness are conveyed and received. We analyzed the top 150 Instagram posts, and this methodological approach ensured a robust analysis of the content and engagement of CRC-related posts on Instagram, providing insights into the effectiveness of social media as a tool for health awareness campaigns. Our findings highlighted the diverse approaches used to raise awareness about CRC on Instagram.

A key finding was the dominance of themes related to screening and early detection, which reflects the goal of Colorectal Cancer Awareness Month to raise awareness about CRC and motivate individuals to participate in screening, and also suggests that public health campaigns are effectively leveraging Instagram to promote these critical aspects of CRC prevention [2,3,15]. The significant engagement metrics, particularly the high number of likes and comments associated with the hashtag #colorectalcancer, suggest that this specific tag resonates well with the Instagram audience. Additionally, the use of multiple hashtags in posts appeared to enhance engagement, suggesting that combining hashtags may increase the visibility and interaction of the posts.

While the themes of general awareness and risk factors were well represented, the relatively limited number of posts addressing symptoms and individual experiences points to potential gaps in the current messaging strategy. Personal stories can be particularly powerful in motivating behavior change and should be utilized more extensively [22-24]. Furthermore, the underrepresentation of posts targeting racial and ethnic minoritized communities and women highlights a critical gap in the current approach. Given the disparities in CRC incidence and outcomes among different demographic groups, it is essential to tailor messages to address these disparities and ensure more inclusive outreach [1,3].

This study supports the existing literature on the effectiveness of visual content in attracting user interaction [10-15]. However, discrepancies may exist regarding the types of messages that are most effective. For instance, while some studies suggest that statistical information is persuasive, the current findings highlight the potential impact of personal narratives, which may be underutilized in existing campaigns [24].

### Implications for Public Health Practice

The results of this study have several implications for public health practice. First, there is a need for more tailored campaigns that specifically address the risks and experiences of marginalized groups and women [25]. Developing culturally sensitive and inclusive content can help bridge the current gaps in outreach [26]. Second, incorporating more individual experiences and testimonies in posts can create more relatable and impactful messages, potentially increasing awareness and encouraging preventive behaviors [27]. Lastly, public health campaigns should consider using multiple and specific hashtags to maximize reach and engagement [28].

### Limitations and Future Research

This study has several strengths, including a comprehensive analysis of the top posts on a significant awareness month and high interrater reliability in content classification, which ensures consistency in the findings. However, there are also limitations. The study is limited to 1 social media platform (Instagram) and 1 specific month, which may not capture the full spectrum of awareness activities and engagement throughout the year on other social media platforms. Instagram was chosen due to its visual-centric nature, which is particularly well suited for health awareness campaigns, and its algorithmic features that facilitate content discovery and engagement through hashtags. Additionally, the overlap between Instagram and Facebook, both owned by Meta, often results in similar campaign content across the 2 platforms, providing a representative snapshot of Meta’s ecosystem. Moreover, the cross-sectional nature of the study limits the ability to assess changes over time or the long-term impact of these posts.

We did not assess the credibility and quality of the Instagram posts, which was a deliberate decision, as the study aimed to explore thematic content and user engagement rather than verify the accuracy or reliability of the posts. The credibility of posts is important for assessing the public health value of messages [29]. Future research should consider assessing the quality and credibility of health-related social media content to better understand its reliability and influence on public health outcomes.

Future research should consider longitudinal studies to evaluate the impact of social media campaigns over time. Comparative studies across different social media platforms would also be beneficial to identify the most effective channels for health promotion. Furthermore, research focusing on the development and testing of tailored messages for underrepresented groups is necessary to enhance the inclusivity and effectiveness of these campaigns. Finally, while this study focused on predefined themes, we recognize the value of inductive approaches, such as topic modeling, for identifying emergent themes or uncovering patterns directly from the data. Future research could complement our findings by employing such methods to explore new dimensions of social media health communication.

### Conclusion

In conclusion, this study highlights the potential of Instagram as a platform for promoting CRC awareness, particularly for screening and early detection. While certain themes effectively engage users and align with the goals of awareness campaigns, critical gaps remain in inclusivity and representation, particularly for marginalized communities. To maximize the impact of future campaigns, messaging strategies should be refined and incorporate a broader range of themes, ensuring that social media platforms like Instagram are leveraged more effectively to raise awareness, reduce disparities, and promote preventive health behaviors.

## Abbreviations

CRC: colorectal cancer
HPV: human papillomavirus
STROBE: Strengthening the Reporting of Observational Studies in Epidemiology
